# The development of practice standards for patient education in nurse-led clinics: a mixed-method study

**DOI:** 10.1186/s12912-023-01444-0

**Published:** 2023-08-22

**Authors:** Zohre Pouresmail, Fatemeh Heshmati Nabavi, Maryam Rassouli

**Affiliations:** 1 Medical-Surgical Nursing Department, School of Nursing and Midwifery, Mashhad University of Medical Sciences, Mashhad, Iran; 2 Nursing and Midwifery Care Research Center, Mashhad University of Medical Sciences, Mashhad, Iran; 3 Department of Community Health and Psychiatric Nursing, School of Nursing and Midwifery, Mashhad University of Medical Sciences, Mashhad, Iran; 4 School of Nursing & Midwifery, Shahid Beheshti University of Medical Sciences, Tehran, Iran

**Keywords:** Practice standards, Patient education, Nurse-led clinic

## Abstract

**Introduction:**

Educating patients and families about self-care is one of the important roles of nurses in Nurse-led clinics (NLCs). NLCs need standards for guiding the practice of nurses. A standard is an authoritative statement that sets out the legal and professional basis of nursing practice. This paper seeks to report the development of practice standards for patient and family education in NLCs.

**Methods:**

This project used a Sequential-Exploratory mixed methods design. Before the study, we conducted a literature review to identify gaps. Directed content analysis was used in phase 1. The second phase involved two focus groups. The third phase involves two rounds of modified Delphi.

**Results:**

Twenty-nine participants were interviewed, and 1816 preliminary codes were formed in phase 1. 95 standards were grouped into three main categories (structure, process, and outcome). In the first focus group, experts eliminate 32 standards. Experts eliminate 8 standards after the second stage of the focus group. After two rounds of Delphi, the final version of the standard consists of 46 standards (13 structure, 28 process and 5 outcome).

**Conclusions:**

Nurses and institutions could benefit from practice standards for patient education in the NLCs, which consist of 46 statements in three domains, as a guide for clinical activities and a tool to gauge the quality of patient education in NLCs. The developed standards in this study can guide new and existing NLCs and help them evaluate ongoing activities. Providing patient education in NLCs based on standards can improve patients’ outcomes and promote their health.

**Supplementary Information:**

The online version contains supplementary material available at 10.1186/s12912-023-01444-0.

## Highlights

• Existence of practice standards for patient education in NLCs is necessary.

• Structure standards necessary for the establishment of NLCs.

• Process standards guide practice in NLCs.

• Outcome standards used for evaluation of NLCs performance.

**Supplementary Information:**

The online version contains supplementary material available at 10.1186/s12912-023-01444-0.

## Background

Nursing has evolved to meet the dynamic needs of individuals, communities, and healthcare services. Aging populations are creating a greater demand for health resources, causing changes in service delivery and higher rates of chronic disease in the community [1]. As health services are increasingly focused on keeping people in their communities and minimizing hospitalizations, Nurse-led Clinics (NLCs) are well-suited to accomplish this goal [2]. At the same time, it has been argued that NLCs can provide cost-effective, high-quality care and improve patient access to services [3]. There is evidence that NLCs improve healthcare, patient, and quality care outcomes [4], patient satisfaction [5], and treatment adherence [6]. These clinics are equipped with nurses who assess, admit, educate, treat, monitor, discharge, and provide the patients with psychological support and refer them to other healthcare professionals [7]. Training and educating patients and families about self-care is one of the important roles of nurses in NLCs. Also, NLCs tend to be specialized [8]. For this purpose, NLCs were established for different diseases such as liver cirrhosis [9], atrial fibrillation [10], ulcer care [11], diabetes [12], thyroid cancer [13], rheumatology [14], heart failure [15], and other chronic diseases. It has been found that NLCs can improve chronic disease management, reduce treatment burden [16], and positively impact patient outcomes such as satisfaction, access to care, and cost-effectiveness [1].

Nurse-led clinics need standards for guiding the practice of nurses. Nursing standards are authoritative statements that outline the legal and professional basis for nursing practice. Safe and effective practice requires knowledge, skills, judgment, and attitudes outlined in all standards of practice. A clinician’s performance, attributes, and expected outcomes are guided by practice standards [17]. The Joint Commission (TJC) delineated nursing standards for patient education as early as 1993. As mandates, these standards describe positive outcomes of patient care. They must be met through teaching activities by nurses in the hospital that must be patient and family-oriented [18]. TJC has established nursing standards for patient education in ambulatory care, home care, and primary care centers. These standards define the performance expectations, structures, or functions that must be in place for an organization to be accredited by TJC [18].

The importance of addressing the educational needs of patients and the impact of education on enhancing patient outcomes, especially for those with chronic conditions and those receiving outpatient care, led to the establishment of independent nurse-led clinics in Iran in 2010. These clinics aim to provide education and counseling services to patients in hospital outpatient wards in major cities across Iran. The provision of services in these centers has been voluntary and creative. Given that the educational role of nurses is expanding, starting and continuing the activities of these clinics has always faced barriers. Some of these barriers include not defining the position of these centers in the hospital organizational structure [19], lack of independence in providing services, and difficulty providing human resources [20]. Some concerns are related to unclear patient education work processes and interdepartmental cooperation, which affect the provision of patient education. Other concerns pertain to the societal culture and the level of trust patients have in nurses to deliver high-quality and reliable education [21]. In this regard, Farahani et al.‘s (2007) study found that the nurses and their roles were not recognized well, and most individuals in society were unaware of nurses’ scientific and practical competencies [22].

Following international trends and the evolution of patient education from hospitals to outpatient centers, as well as home and community care, the development and promotion of patient and family education programs became a research priority for the Nursing Deputy of the Ministry of Health in 2019. In June 2022, the Nursing Deputy of the Ministry of Health, Treatment, and Medical Education officially announced the “executive instruction of nurse-led clinics for patient education and follow-up” to the entire country [23].

For this newly developed service and its standards to perform perfectly in implementation and evaluation, it should be explained based on one of the quality evaluation models. Donabedian’s (1966) Structure-Process-Outcomes (SPO) conceptual framework was used to examine health services and evaluate the quality of care. The model comprises three elements. The structure is described as the setting in which care is delivered that encompasses resources, quality client care standards, staffing, policies, and structural elements that lay a foundation for quality healthcare services. The process focuses on how things work within an organization and the framework that guides the design of the organization. Processes define the mechanisms for producing intended outcomes and include continuity of care, professional models of care delivery, and interpersonal management of patient care. The outcomes focus on client status after healthcare delivery, including client knowledge and behavior, patient satisfaction, and health-related quality of life [24]. All three elements of Donabedian’s framework must be in place and monitored for quality to occur. A good structure increases the likelihood of good processes that can ultimately result in good outcomes [25–27]. Organizations and professions must set standards and objectives to provide safe and effective care [28]. Nurses need to set standards for patient education in this new setting.

### Aim

This paper reports the development of practice standards for patient education in NLCs in three phases.

#### Phase 1

Developing patient education standards for NLCs.

#### Phase 2

Validation of Practice standards for patient education in NLCs from perspective of experts.

#### Phase 3

Determining the agreement, appropriatness, relevance and clarity of practice standards for patient education in NLCs from the perspective of experts.

## Methods

### Design

**Fig. 1:**
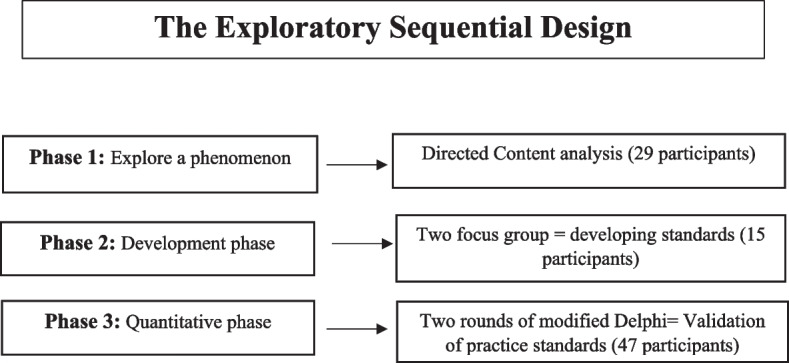
The exploratory sequential design

This study used a sequential exploratory mixed-method design [29] (Fig. 1). Before the study, we conducted a literature review to identify the gaps. We did not find practice standards for patient education in NLCs, but we found patient and family education standards and used them to develop practice standards. Phase 1 involved a qualitative study using directed content analysis based on Assarroudi et al. (2018) [30]. We performed content analyses in three main phases: Preparation, organizing, and reporting [31]. Based on Asarroodi et al.’s (2018) inductive content analysis method, the preparation phase was performed by going through seven stages including acquiring the necessary general skills, selecting the appropriate sampling strategy, deciding on the analysis of manifest and/or latent content, developing an interview guide, conducting interviews and transcribing interviews, specifying the units of analysis, and being immersed in the data [30]. At this stage, after transcribing each interview and considering its transcribed text as the unit of analysis, each text was read several times until the data immersion occurred. During this stage, the answer to these questions was always taken into consideration by the researcher: What event is happening? Who is speaking? Where is it happening? When did it happen? What is happening and why?.

Based on Asarroodi et al.’s (2018) content analysis method, the organizing phase consisted of developing a formative categorization matrix, the theoretical definition of the main category and subcategories, determining coding rules for the main category, pre-testing the categorization matrix, choosing and specifying the anchor samples for each main category, performing the main data analysis, the inductive abstraction of the main categories from preliminary codes, and establishing links between the generic categories and main categories [30, 32]. The researchers, in the organizing phase, created a constrained matrix for analysis based on Structure-Process- Outcome Donabedian’s model. In this matrix, the creation of new main categories is not allowed. The data were reviewed several times to find content that matched predefined categories or could be a sample for them, and preliminary codes were assigned to them. Afterward, the stages of grouping, categorization, and abstraction were performed so that the generic categories were created, and the possibility of placing these generic categories in the main categories in the matrix was then examined conceptually and logically [33].

Phase 2 involved two focus groups, and Phase 3 consisted of two rounds of modified Delphi [34]. The Ethics Committee approved this study. All participants in the study signed an informed consent form.

### Eligibility

#### Phase 1: Directed Content Analysis (DCA)

In phase 1, three groups of participants were eligible to participate. The first group consisted of hospital managers and supervisors, the second group comprised physicians and nurses, and the third group included patients and their caregivers.

#### Phases 2 and 3: focus group and modified Delphi

The eligibility criteria for phases 2 (focus group) and 3 (modified Delphi) included (a) faculty members and policymakers in patient education, (b) managers and policymakers in patient education, (c) physicians participating in patient education planning, (d) nurses participating in patient education planning, (e) nursing faculty members designing and editing patient education content or authoring a book on patient education, and (f) health education supervisors with at least one year of experience in their position.

### Study setting

This study was performed at the Mashhad University of Medical Sciences and the Deputy Minister of Nursing, Ministry of Health, Treatment, and Medical Education. Mashhad is one of Iran’s largest and leading cities conducting patient education programs.

### Sampling and sample size

#### Phase 1: directed content analysis

A purposive sampling method was used for sampling, which continued until data saturation. Group 1 consisted of 4 educational supervisors, 4 health education supervisors (the health education supervisor and educational supervisor are the middle managers responsible for designing, implementing, and supervising educational programs for staff, patients, and clients), 2 nursing managers, 2 chief executive officers, and 1 deputy medical specialist. Group 2 consisted of 5 nurses, 3 doctors, and 2 nursing faculty members, and group 3 consisted of 4 patients and 2 patient caregivers.

#### Phase 2: focus groups

There are no universally accepted criteria for selecting experts in focus groups [35]. A multi-professional panel was created with faculty members having adequate experience as a member of a patient education or policy-making team in patient education. Thus, we invited 15 nursing faculty members and policymakers from Iran’s Ministry of Health, Treatment, and Medical Education.

#### Phase 3: modified Delphi

There were two Delphi phase rounds, each lasting four weeks with four-week intervals. Non-respondents received weekly e-mail reminders. We did not provide any financial incentives. Based on Drisko, quoted from Wellar (2008), a panel of fewer than 10 people provides diversity in expert opinions, and Jones and Twiss (1978) recommend 10 to 50 participants [36]. Therefore, 47 nursing and policy-making experts participated in this phase. We mailed each panelist a questionnaire outlining patient education standards during the first survey round. Using a five-point Likert scale, each member rated their agreement with each standard: (1) Strongly disagree, (2) disagree, (3) neither agree nor disagree, (4) agree, and (5) strongly agree [37]. We asked the participants to provide a reason for each disagreement. A consensus was defined a priori in this study when at least 80% of the experts agreed. First-round survey results were sent to the research team, and disagreements were discussed. To conduct the second round of surveys, we mailed questionnaires to each panelist indicating the standard of patient education. Based on a nine-point Likert scale, each member rated each statement’s relevancy, appropriateness, and clarity (1–3 inappropriate, 4–6 intermediate, and 7–9 appropriate).

### Statistical analysis

All statistical analyses were done using the SPSS software package, version 11.0.

## Results

### Participant characteristics

Table 1 summarizes the characteristics of participants in the three phases of the study. All nurses and experts in this study had experience in patient education. Among 47 experts surveyed, 40 (85.1%) responded in over two rounds.

**Table 1:** Characteristics of the participant in 3 phases of the study

Phase/step	participant	Age (mean SD)	Work experience (mean SD)	Gender N (%)	Educational level N (%)	Employment classification N (%)
**Phase 1; Directed Content Analysis**	**Group1** (Hospital managers and supervisors)	44.84 ± 5.53	5.5 ± 1.83	Female: 10 (76.9) Male: 3 (23.1)	Master: 11 (84.6) doctorial: 2 (15.4)	Educational supervisors: 4 (30.8) Health education supervisors: 4 (30.8) Nursing managers: 2 (15.4) Chief Executive Officer: 2 (15.4) Deputy Medical Specialist: 1 (7.7)
	**Group 2** (physicians and nurses)	45.40 ± 8.16	4.2 ± 2.44	Female: 9 (90.0) Male: 1 (10.0)	License: 4 (40.0) Master: 2 (20.0) doctorial: 4 (40.0)	Nurses: 5 (50.0) Physician: 3 (30.0) Nursing faculty member: 2 (20.0)
	**Group3** (patients and their caregivers)	41.33 ± 8.54	Not applicable	Female: 6 (100.0)	Elementary: 3 (50.0) Diploma: 3 (50.0)	Patients: 4 (66.7) Patient’s family: 2 (33.3)
**Phase 2; Focus group**		43.20 ± 5.35	12.2 ± 2.34	Female: 19 (95.0) Male: 1 (5.0)	Master: 9 (45.0) Ph.D.: 11 (55.0)	Faculty member: 11 (55.0) Deputy Medical Specialist: 9 (45.0)
**Phase 3; 2 round of Delphi**		44.02 ± 5.49	10.56 ± 1.42	Female: 32 (80.0) Male: 8 (20.0)	Bachelor: 3 (7.5) Master: 16 (40.0) Ph.D.: 21 (52.5)	Faculty member: 21 (52.5) Nurse: 6 (15.0) Educational supervisor: 1 (2.5) Heath educational supervisor: 9 (22.5) Nursing Director: 3 (7.5)

In the initial review, we did not find specific standards for patient education in NLCs. Facilities and staff were among the reviewed standards in the structure dimension for other healthcare centers (hospitals, home care, and ambulatory care setting). Preliminary assessment, the target group of education, determining and prioritizing the learning needs, the content of patient education, methods and conditions of education, designing programs and materials for patient education, patient participation in education, and referral to specialized organizations were mentioned in the process dimension. The evaluation of educational programs, materials, and learners was mentioned in the outcome dimension (Table 2).

**Table 2:** Patient and family education categories based on review and directed content analysis

Dimensions	Review	Directed content analysis
**Structure**	• Facilities • staff	• Equipment • Facilities • Staff • specifications of the clinic environment • organizational communications • Nursing characteristics
**Process**	• preliminary assessment • the target group of education • determining and prioritizing the learning needs • the content of patient education • methods and conditions of education • designing programs and materials for patient education • patient participation in education • referral to specialized organizations	• Content of patient education • the target group of education • nurse job description • training method • referral form • method of determining patients ‘educational priorities • referral of patients to the clinic • process of preparation educational pamphlet • patient education expenditure • patient follow-up • physicians’ cooperation and promotion performance of the Clinic
**Outcome**	• evaluation of educational programs and materials • learner evaluation	• evaluation of educational programs and materials • learner evaluation

### Results of phase 1: directed content analysis

In phase 1, 29 participants were interviewed, and 1,816 preliminary codes emerged. Content analysis was performed based on Assarroudi et al. (2018). Donabedian’s model was used as the data analysis framework. The structure’s main category included six generic categories (equipment, facilities, staff, specifications of the clinic environment, organizational communications, and nursing characteristics). Also, 12 generic categories were found in the process main category (content of patient education, target group of education, nurse job description, training method, referral form, method of determining patients’ educational priorities, referral of patients to the clinic, process of educational pamphlet preparation, patient education expenditure, patient follow-up, physicians’ cooperation, and promotion performance of the clinic). There was one generic category (i.e., evaluation) in the outcome main category (Table 2). Data comparison was made at the end of this phase to compare data from different sources [29]. Based on data comparison, we developed 15 standards in the structure (7 standards based on DCA, 7 based on review, and one standard based on review and DCA), 73 standards in the process (15 standards based on review, 27 standards based on DCA, and 31 standards based on review and DCA), and 7 standards in the outcome (5 standards based on DCA and two standards based on review).

### Results of phase 2: two rounds of focus group

#### Step 1

In the focus group, experts eliminated standards related to patient education during hospitalization. At the end of this session, 13 standards in the structure, 43 standards in process, and 7 standards in the outcome remained.

#### Step 2

Before this session, the standards were sent to the participants for review and comment. Based on expert opinions, some standards were written and revised entirely. Also, 9 process standards were not agreed upon by the experts and were removed and merged with other standards. After the focus group, 13 standards in the structure, 37 in the process, and 5 in the outcome remained.

### Results of phase 3: two rounds of modified Delphi

#### Step 1

At this stage, experts’ agreement with the standards was determined. Development standards were sent to 47 experts in nursing and policy-making. During the first round of the survey, 40 panelists responded; 46 statements (83.63%) were judged appropriate by more than 80% of the respondents, and 9 statements (16.36%) were disagreed upon (Table 3). According to the experts, some standards were completely rewritten, especially in the process domain. Based on the experts, 13 standards in the structure, 28 in the process, and 5 in the outcome remained.

**Table 3:** Experts’ agreement about developed standards for patient education in NLCs

Domain	Standards	Agreement			Disagreement		Agreement percent
		5	4	3	2	1	
Structure	**Standard 1**: The head and director of the hospital, the director of nursing, the health education supervisor, and the head nurse of the clinic cooperate in establishing and supervising the Nurse-led clinic (NLC).	27 (77.1)	6 (17.1)	-	2 (2.9)	-	**33 (94.2)**
	**Standard 2**: The patient education committee in the hospital has been formed with the participation of the head and director of the hospital, the nursing director, the health education supervisor, the head nurse of the clinic, and educating nurses in the NLC.	25 (71.4)	8 (22.9)	-	1 (2.9)	-	33 (94.3)
	**Standard 3**: The hospital has defined the mechanism of interdisciplinary cooperation in patient and family education in the NLC.	27 (77.1)	5 (14.3)	-	2 (5.7)	-	32 (91.4)
	**Standard 4**: The hospital has specified and announced the role and duties of the nurse, physician, and non-professional staff of the clinic (secretary, guard, etc.) regarding the activities of the health education nursing clinic.	23 (65.7)	6 (17.1)	2 (5.7)	3 (8.6)	-	29 (82.8)
	**Standard 5**: A job description for the educating nurse in NLC is exist and available.	25 (71.4)	5 (14.3)	2 (5.7)	2 (5.7)	-	30 (85.7)
	**Standard 6**: The hospital provides counseling services for nursing educators in patient education (the possibility of contacting and consulting with medical and nursing professors, books, and updated print and online instructions) to answer patients’ questions.	25 (71.4)	5 (14.3)	2 (5.7)	2 (5.7)	-	30 (85.7)
	**Standard 7**: The hospital has provided the possibility of participating nursing educators in the NLC in codified patient education courses, health literacy, self-care, and self-management.	25 (71.4)	7 (20.0)	-	2 (5.7)	-	32 (91.4)
	**Standard 8**: The hospital selects educating nurses in the NLC based on their competencies	26 (74.3)	6 (17.1)	-	2 (5.7)	-	32 (91.4)
	**Standard 9**: The hospital selects the educating nurses in the NLC based on their meta-competencies.	28 (80.0)	2 (5.7)	1 (2.9)	1 (2.9)	2 (5.7)	30 (85.7)
	**Standard 10**: The hospital provides the standard physical environment for the NLC.	25 (71.4)	7 (20.0)	1 (2.9)	1 (2.9)	-	32 (91.4)
	**Standard 11**: The hospital provides training equipment, facilities, and educational assistance tools based on patients’ and their families’ educational needs and preferences.	27 (77.1)	5 (14.3)	-	1 (2.9)	-	32 (91.4)
	**Standard 12**: The hospital has provided the necessary facilities for patients to access the NLC.	27 (77.1)	5 (14.3)	-	1 (2.9)	-	32 (91.4)
	**Standard 13**: In the operational planning of the hospital, planning has been done for the development of training and counseling services in the NLC.	27 (77.1)	5 (14.3)	-	1 (2.9)	-	32 (91.4)
Process	**Standard 1**: The target group of patient education in NLCs is determined based on the type of disease and the number of patients referred to the hospital’s outpatient clinics.	14 (40.0)	13 (37.1)	3 (8.6)	3 (8.6)	1 (2.9)	27 (77.1)
	**Standard 2**: The hospital uses the referral form to refer patients from the physician and inpatient wards to the NLCs.	20 (57.1)	7 (20.0)	4 (11.4)	3 (8.6)	-	27 (77.1)
	**Standard3**: The hospital plans to improve the performance of the NLCs in serving clients and the community (improving the number of referring patients).	23 (65.7)	9 (25.7)	1 (2.9)	1 (2.9)	-	32 (91.4)
	**Standard 4**: The nurse, if necessary, refers the patient to the NLCs in specialized hospitals and related social organizations.	19 (54.3)	10 (28.6)	1 (2.9)	4 (11.4)	-	29 (82.9)
	**Standard 5**: The working hours of the NLCs should be daily and regular, preferably during the attendance hours of the hospital clinic physicians.	23 (65.7)	4 (11.4)	1 (2.9)	5 (14.3)	1 (2.9)	27 (76.8)
	**Standard5**: nurses in the NLCs work based on their job descriptions.	14 (40.0)	13 (37.1)	3 (8.6)	3 (8.6)	1 (2.9)	27 (77.1)
	**Standard 7**: Planning the performance of the NLCs as a team in the hospital and coordination with the Vice-Chancellor of the University, taking into account the specialty of the hospital, the number of patients referred to the hospital clinic, and the attendance plan of physicians	15 (42.9)	10 (28.6)	4 (11.4)	4 (11.4)	1 (2.9)	25 (71.5)
	**Standard 8**: The University Vice-Chancellor is responsible for overseeing the establishment and operation of NLCs in hospitals.	23 (65.7)	7 (20.0)	1 (2.9)	2 (5.7)	1 (2.9)	30 (85.7)
	**Standard 9**: The hospital performs its duties in the field of setting up and operating NLCs.	22 (62.9)	7 (20.0)	1 (2.9)	3 (8.6)	-	29 (82.9)
	**Standard 10**: The hospital director and manager use appropriate methods to engage physicians to refer patients to Ns.	19 (54.3)	8 (22.9)	1 (2.9)	4 (11.4)	1 (2.9)	27 (77.2)
	**Standard 11**: program and training materials (annual) should be reviewed.	30 (85.7)	3 (8.6)	-	1 (2.9)	-	33 (94.3)
	**Standard 12**: The hospital has determined the cost of patient education.	19 (54.3)	7 (20.0)	1 (2.9)	3 (8.6)	3 (8.6)	26 (74.3)
	**Standard 13**: Needs assessment and training priorities for patients referred to the NLCs are performed at appropriate intervals in the hospital.	20 (57.1)	11 (31.4)	2 (5.7)	1 (2.9)	-	31 (88.5)
	**Standard 14**: Learning Objectives for Patient Education in the NLCs are set by the care team in a codified educational program.	22 (62.9)	7 (20.0)	2 (5.7)	1 (2.9)	2 (5.7)	29 (82.9)
	**Standard 15**: Develop an educational program with a precise definition of behavioral and educational goals for groups of patients.	21 (60.0)	6 (17.1)	5 (14.3)	1 (2.9)	1 (2.9)	27 (77.1)
	**Standard 16**: The content of patient education is prepared based on a well-designed program in the hospital, educational goals, target group and, the group needs assessment.	27 (77.1)	6 (17.1)	1 (2.9)	-	-	33 (94.2)
	**Standard 17**: Nurses provide appropriate training materials to patients to complete their training.	25 (71.4)	7 (20.0)	-	1 (2.9)	-	32 (91.4)
	**Standard 18**: Patient education record (needs assessment, inclusive, education method, duration of education, feedback received from education) is recorded in the education form.	25 (71.4)	7 (20.0)	1 (2.9)	1 (2.9)	-	32 (91.4)
	**Standard 19**: Patient education documentation must be accurate, clear and legal.	26 (74.3)	6 (17.1)	-	2 (5.7)	-	32 (91.4)
	**Standard 20**: Evaluation of training programs must be accurate and clear.	27 (77.1)	4 (11.4)	1 (2.9)	-	-	31 (88.5)
	**Standard 21**: Codified training programs are evaluated annually.	23 (65.7)	8 (22.9)	1 (2.9)	2 (5.7)	-	31 (88.6)
	**Standard 22**: patient education working group/committee prioritizes follow-up for patients.	16 (45.7)	12 (34.3)	4 (11.4)	-	1 (2.9)	28 (80.0)
	**Standard 23**: The Patient Education Working Group / Committee plans and acts to follow patients.	18 (51.4)	12 (34.3)	3 (8.6)	-	-	30 (85.7)
	**Standard 24**: Patient education needs assessment is performed and recorded by the nurse for each patient based on the educational needs assessment.	21 (60.0)	7 (20.0)	3 (8.6)	2 (5.7)	1 (2.9)	28 (80.0)
	**Standard 25**: Patient education is prioritized based on individual needs assessment and a well-designed program.	23 (65.7)	8 (22.9)	3 (8.6)	-	-	31 (88.6)
	**Standard 26**: Teaching patients is a combination of face-to-face and absentee methods, taking into account the preferences of patients and families.	23 (65.7)	11 (31.4)	-	-	-	34 (97.1)
	**Standard 27**: Patient education is done as a team with the participation of all caring team members in education.	20 (57.1)	7 (20.0)	4 (11.4)	3 (8.6)	-	27 (77.1)
	**Standard 28**: Patient education is based on respect for patient privacy, confidentiality, and respect for patients’ values ​​and beliefs.	30 (85.7)	4 (11.4)	-	-	-	34 (97.1)
	**Standard 29**: Patient education should be tailored to the patient’s condition, for the patient’s time, as soon as possible, by the patient’s physical condition, and when they can concentrate.	26 (74.3)	7 (20.0)	-	-	1 (2.9)	33 (94.3)
	**Standard 30**: The duration of patient education in the health education clinic is determined depending on the patient’s condition.	28 (80.0)	4 (11.4)	1 (2.9)	-	-	32 (91.4)
	**Standard 31**: There is evidence that the patient and family are involved in determining educational needs and choosing teaching methods.	23 (65.7)	8 (22.9)	1 (2.9)	1 (2.9)	1 (2.9)	31 (88.6)
	**Standard 32**: Patient’s understanding of education is assessed in the NLCs using patient questioning, observation and return-demonstration methods.	25 (71.4)	9 (25.7)	-	-	-	34 (97.1)
	**Standard 33**: Patient perception of education is reviewed and recorded at the end of the training session.	26 (74.3)	8 (22.9)	-	-	-	34 (97.2)
	**Standard 34**: The hospital has developed an appropriate process and protocol for preparing, distributing and storing educational materials (pamphlets, multimedia).	25 (71.4)	7 (20.0)	2 (5.7)	-	-	32 (91.4)
	**Standard 35**: The hospital uses the appropriate process to prepare standard educational materials for compiling educational content.	22 (62.9)	9 (25.7)	1 (2.9)	-	1 (2.9)	31 (88.6)
	**Standard 36**: Various methods of distributing educational materials according to hospital conditions and patients’ preferences are used (electronic and print distribution).	24 (68.8)	5 (14.3)	2 (5.7)	1 (2.9)	1 (2.9)	29 (83.1)
	**Standard 37**: The hospital uses appropriate training materials to educate patients in the NLCs.	25 (71.4)	6 (17.1)	2 (5.7)	-	-	31 (88.5)
Outcome	**Standard 1**: Patients referred to the NLC know the risk factors for chronic diseases, complications and prevention methods.	25 (71.4)	7 (20.0)	-	1 (2.9)	-	32 (91.4)
	**Standard 2**: Patients referred to the NLC know ways to improve and maintain a healthy lifestyle.	24 (68.6)	7 (20.0)	-	1 (2.9)	1 (2.9)	31 (88.6)
	**Standard 3**: Referrals to the NLC make informed decisions to control their illness and lead a healthy lifestyle based on cultural and religious values ​​and socioeconomic status.	21 (60.0)	9 (25.7)	1 (2.9)	1 (2.9)	1 (2.9)	30 (85.7)
	**Standard 4**: The physical, mental and emotional health of patients referred to the NLC is promoted.	26 (74.3)	5 (14.3)	1 (2.9)	1 (2.9)	-	31 (88.6)
	**Standard 5**: The hospital examines the short-term and long-term consequences of providing education and counseling services to patients and their families.	24 (68.6)	8 (22.9)	-	1 (2.9)	-	32 (91.5)

#### Step 2

All panelists responded in the second round; 46 statements (100%) were relevant, appropriate, and clear (Table 4). The final standard inventory consisted of 46 statements (13 in the structure, 28 in the process, and 5 in the outcome; Additional file 1).

**Table 4:** Experts’ openion about appropriateness, clarity and relevancy of developed standards for patient education in NLCs

Domain			Standards	Relevancy				Appropriatness				Clarity			
				7–9 Relevant N (%)	4–6 Partial relevant N (%)	1–3 Non relevant N (%)	Total N (%)	7–9 Appropriat N (%)	4–6 partial Appropriate N (%)	1–3 Non appropriat N (%)	Total N (%)	7–9 Clear N (%)	4–6 Patialy clear N(%)	1–3 Non clear N (%)	Total N (%)
**Structure**	**team/ teamwork**		**Standard 1**: The head and director of the hospital, the director of nursing, the health education supervisor, and the head nurse of the clinic cooperate in establishing and supervising the Nurse-led Clinic (NLC).	39 (97.5)	0	0	39 (97.5)	39 (97.5)	0	0	39 (97.5)	37 (92.5)	2 (5.0)	1 (2.5)	40 (100)
			**Standard 2**: The patient education committee in the hospital has been formed with the participation of the head and director of the hospital, the nursing director, the health education supervisor, the head nurse of the clinic, and educating nurses in the NLC.	40 (100)	0	0	40 (100)	39 (97.5)	0	0	39 (97.5)	37 (92.5)	2 (5.0)	1 (2.5)	40 (100)
			**Standard 3**: The hospital has defined the mechanism of interdisciplinary cooperation in patient and family education in the NLC.	40 (100)	0	0	40 (100)	39 (97.5)	0	0	39 (97.5)	36 (90.0)	2 (5.0)	2 (5.0)	40 (100)
			**Standard 4**: The hospital has specified and announced the role and duties of the nurse, physician, and non-professional staff of the clinic (secretary, guard, etc.) regarding the activities of the NLC.	38 (95.0)	0	1 (2.5)	39 (97.5)	35 (87.5)	2 (5.0)	1 (2.5)	38 (95.0)	35 (87.5)	4 (10.0)	1 (2.5)	40 (100)
			**Standard 5**: A job description is exist and available for the educating nurse in the NLC.	39 (97.5)	0	1 (2.5)	40 (100)	38 (95.0)	1 (2.5)	0	39 (97.5)	37 (92.5)	2 (5.0)	1 (2.5)	40 (100)
	**Advisory Committee**		**Standard 6**: The hospital provides counseling services for nursing educators in patient education (the possibility of contracting and consulting with medical and nursing professors, books, and updated print and online instructions) to answer patients’ questions.	37 (92.5)	1 (2.5)	1 (2.5)	39 (97.5)	37 (92.5)	2 (5.0)	0	39 (97.5)	34 (85.0)	4 (10.0)	2 (5.0)	40 (100)
	**Professional skills and continuing education**		**Standard 7**: The hospital has provided the possibility of participating nursing educators in the NLC in codified patient education courses, health literacy, self-care, and self-management.	40 (100)	0	0	40 (100)	38 (95.0)	1 (2.5)	0	39 (97.5)	37 (92.5)	3 (7.5)	0	40 (100)
			**Standard 8**: The hospital selects educating nurses in the NLC based on their competencies.	40 (100)	0	0	40 (100)	36 (90.0)	3 (7.5)	1 (2.5)	40 (100)	37 (92.5)	2 (5.0)	1 (2.5)	40 (100)
			**Standard 9**: The hospital selects the educating nurses in the NLC based on their meta-competencies.	38 (95.0)	0	0	38 (95.0)	35 (87.5)	3 (7.5)	1 (2.5)	39 (97.5)	38 (95.0)	0	1 (2.5)	39 (97.5)
	**Physical space and equipment, Facilities, and planning**		**Standard 10**: The hospital provides the standard physical environment for the NLC.	40 (100)	0	0	40 (100)	40 (100)	0	0	40 (100)	38 (95.0)	1 (2.5)	1 (2.5)	40 (100)
			**Standard 11**: The hospital provides training equipment, facilities, and educational assistance tools based on patients’ and their families’ educational needs and preferences.	39 (97.5)	0	0	39 (97.5)	40 (100)	0	0	40 (100)	37 (92.5)	1 (2.5)	1 (2.5)	39 (97.5)
			**Standard 12**: The hospital has provided the necessary facilities for patients to access the NLC.	39 (97.5)	0	0	39 (97.5)	36 (90.0)	3 (7.5)	1 (2.5)	40 (100)	34 (85.0)	3 (7.5)	2 (5.0)	39 (97.5)
			**Standard 13**: In the hospital’s operational plan, planning has been done to develop training and counseling services in the NLC.	39 (97.5)	0	0	39 (97.5)	38 (95.0)	1 (2.5)	0	39 (97.5)	38 (95.0)	1 (2.5)	1 (2.5)	40 (100)
**Process**	**1. Organizational processes**	**patient education**	**Standard 1**: The hospital determines the components of the patient education process, including needs assessment, planning, implementation, and evaluation of education.	36 (90.0)	2 (5.0)	1 (2.5)	39 (97.5)	38 (95.0)	1 (2.5)	1 (2.5)	40 (100)	34 (85.0)	3 (7.5)	2 (5.0)	39 (97.5)
		**Audience training and referral**	**Standard 2**: Recipients of services in the NLC are determined based on the type of disease and the number of patients referred to hospital outpatient clinics.	37 (92.5)	2 (5.0)	1 (2.5)	40 (100)	35 (87.5)	4 (10.0)	1 (2.5)	40 (100)	37 (92.5)	2 (5.0)	1 (2.5)	40 (100)
			**Standard 3**: The hospital uses appropriate and effective methods to introduce the services of the NLC, identify patients needing training and counseling, and refer them to the NLC.	37 (92.5)	3 (7.5)	0	40 (100)	38 (95.0)	2 (5.0)	0	40 (100)	37 (92.5)	3 (7.5)	0	40 (100)
			**Standard 4**: If necessary, educating nurses in the NLC and considering the patient and family preferences refers them to the NLC in specialized and sub-specialized hospitals and related social organizations.	35 (87.5)	3 (7.5)	1 (2.5)	39 (97.5)	37 (92.5)	2 (5.0)	1 (2.5)	40 (100)	35 (87.5)	3 (7.5)	1 (2.5)	39 (97.5)
		**Clinic activity time**	**Standard 5: T**he hospital provides patients with access to the education and counseling services of the NLC at the appropriate time with a minimum increase in waiting time and in an appropriate manner.	38 (95.0)	1 (2.5)	0	39 (97.5)	37 (92.5)	3 (7.5)	0	40 (100)	39 (97.5)	0	0	39 (97.5)
		**Decision making, and problem-solving**	**Standard 6**: The hospital plans to improve the quantity and quality of services in the NLC.	36 (90.0)	1 (2.5)	1 (2.5)	38 (95.0)	35 (87.5)	3 (7.5)	1 (2.5)	39 (97.5)	35 (87.5)	3 (7.5)	0	38 (95.0)
			**Standard 7**: Education and counseling services are planned and implemented based on the type of the hospital in which lifestyle-related chronic diseases (coronary artery disease, hypertension, diabetes, and cancer) have a priority for the elderly, pregnant women, and children.	34 (85/0)	2 (5.0)	3 (7.5)	39 (97.5)	32 (80.0)	5 (12.5)	2 (5.0)	38 (95.0)	32 (80.0)	5 (12.5)	1 (2.5)	38 (95.0)
			**Standard 8**: Based on a pre-designed operational plan, the activities of the NLC are performed and monitored.	35 (87.5)	2 (5.0)	2 (5.0)	39 (97.5)	35 (87.5)	3 (7.5)	2 (5.0)	40 (100)	38 (95.0)	1 (2.5)	0	39 (97.5)
			**Standard 9**: The hospital provides appropriate facilities and incentives to encourage patients and their families to visit the NLC.	36 (90.0)	2 (5.0)	1 (2.5)	39 (97.5)	36 (90.0)	1 (2.5)	1 (2.5)	38 (95.0)	33 (82.5)	2 (5.0)	2 (5.0)	37 (92.5)
			**Standard 10**: The hospital supports creative and innovative methods to remove barriers to patient education in the NLC.	37 (92.5)	2 (5.0)	1 (2.5)	40 (100)	38 (95.0)	1 (2.5)	1 (2.5)	40 (100)	36 (90.0)	1 (2.5)	2 (5.0)	39 (97.5)
	**2. Group processes**	**Needs assessment**	**Standard 11**: The needs and educational priorities of patients referred to the NLC are properly determined at appropriate intervals in the hospital.	38 (95.0)	1 (2.5)	0	39 (97.5)	40 (100)	0	0	40 (100)	39 (97.5)	0	0	39 (97.5)
		**Determining learning objectives and designing a patient education program**	**Standard 12**: A codified educational program for common diagnoses referred to as the NLC is planned, implemented, and evaluated with a precise definition of learning objectives and training schedules.	38 (95.0)	1 (2.5)	1 (2.5)	40 (100)	36 (90.0)	3 (7.5)	1 (2.5)	40 (100)	38 (95.0)	0	1 (2.5)	39 (97.5)
		**Educational content and use of educational materials**	**Standard 13**: Patients and families are educated concerning specific care measures, nutrition and diet therapy, drug use, self-care training, joint care, physical activity, disease screening, mental health, personal hygiene, maternal health, smoking cessation, rehabilitation, safe use of the equipment, and home safety.	39 (97.5)	1 (2.5)	0	40 (100)	40 (100)	0	0	40 (100)	38 (95.0)	2 (5.0)	0	40 (100)
			**Standard 14**: Educating nurses use appropriate educational materials (pamphlets, posters, videos, pictures, diagrams, and forms during discharge) to educate and strengthen patient and family learning based on their needs and preferences.	37 (92.5)	1 (2.5)	1 (2.5)	39 (97.5)	38 (95.0)	2 (5.0)	0	40 (100)	34 (85.0)	5 (12.5)	0	39 (97.5)
		**Recording patient education**	**Standard 15**: The hospital uses the appropriate methods to record patient education reports (including learning needs, learners, training method, duration of the training, and feedback upon training).	37 (92.5)	1 (2.5)	1 (2.5)	39 (97.5)	38 (95.0)	1 (2.5)	0	39 (97.5)	36 (90.0)	2 (5.0)	0	38 (95.0)
			**Standard 16**: Recording patient education should be accurate, precise, and legal.	38 (95.0)	2 (5.0)	0	40 (100)	39 (97.5)	1 (2.5)	0	40 (100)	37 (92.5)	2 (5.0)	1 (2.5)	40 (100)
		**evaluation**	**Standard 17**: The hospital evaluates NLC programs and reports their results to stakeholders.	39 (97.5)	1 (2.5)	0	40 (100)	39 (97.5)	0	0	39 (97.5)	38 (95.0)	1 (2.5)	0	39 (97.5)
			**Standard 18**: Codified training programs are reviewed at appropriate intervals.	39 (97.5)	1 (2.5)	0	40 (100)	40 (100)	0	0	40 (100)	39 (97.5)	1 (2.5)	0	40 (100)
			**Standard 19**: The hospital plans and takes corrective measures to evaluate the quality and appropriateness of educational materials (educational texts and videos).	39 (97.5)	1 (2.5)	0	40 (100)	39 (97.5)	0	0	39 (97.5)	36 (90.0)	3 (7.5)	0	39 (97.5)
		**patient Follow-up**	**Standard 20**: The hospital plans to provide post-discharge education and follow-up services for at least three groups of priority patients.	38 (95.0)	1 (2.5)	1 (2.5)	40 (100)	39 (97.5)	0	0	39 (97.5)	34 (85.0)	4 (10.0)	0	38 (95.0)
	**3. Individual training processes**	**Needs assessment and training prioritization**	**Standard 21**: The educating nurse conducts a needs assessment, determines the patient and family’s readiness and learning styles, and records the results.	39 (97.5)	1 (2.5)	0	40 (100)	38 (95.0)	1 (2.5)	0	39 (97.5)	36 (90.0)	1 (2.5)	2 (5.0)	39 (97.5)
			**Standard 22**: Educational needs are prioritized based on individual needs assessment and a well-designed program.	35 (87.5)	4 (10.0)	0	39 (97.5)	36 (90.0)	3 (7.5)	0	39 (97.5)	33 (82.5)	5 (12.5)	0	38 (95.0)
		**method and duration of the training**	**Standard 23**: Teaching patients in the NLC involves the combination of face-to-face and online methods, considering patients’ and families’ facilities, values ​​, and preferences.	36 (90.0)	3 (7.5)	0	39 (97.5)	38 (95.0)	2 (5.0)	0	40 (100)	37 (92.5)	2 (5.0)	0	39 (97.5)
			**Standard 24**: Patient education in the NLC includes privacy, confidentiality, and respect for patient’s values ​​and opinions.	39 (97.5)	1 (2.5)	0	40 (100)	38 (95.0)	1 (2.5)	0	39 (97.5)	39 (97.5)	1 (2.5)	0	40 (100)
			**Standard 25**: Patient education in the NLC is planned and implemented as soon as possible, considering the patient’s physical condition and avoiding wasting time.	38 (95.0)	1 (2.5)	1 (2.5)	40 (100)	39 (97.5)	1 (2.5)	0	40 (100)	38 (95.0)	2 (5.0)	0	40 (100)
		**Evaluate patient education**	**Standard 26**: At the end of the training session, the teaching nurse assesses the understanding of the patient and the family from the education by questioning the patients and observation and representation methods and records the results in the patient education registration form in the NLC or the patient’s electronic file.	39 (97.5)	1 (2.5)	0	40 (100)	40 (100)	0	0	40 (100)	39 (97.5)	1 (2.5)	0	40 (100)
		**The process of preparing educational content**	**Standard 27**: The hospital plans to prepare and update appropriate educational materials with quality based on the needs and preferences of patients.	38 (95.0)	1 (2.5)	1 (2.5)	40 (100)	39 (97.5)	1 (2.5)	0	40 (100)	37 (92.5)	1 (2.5)	1 (2.5)	39 (97.5)
			**Standard 28**: The hospital uses appropriate methods for distributing and storing educational content and materials (pamphlets and multimedia).	39 (97.5)	1 (2.5)	0	40 (100)	40 (100)	0	0	40 (100)	38 (95.0)	1 (2.5)	0	39 (97.5)
**Outcome**	**Primary prevention**		**Standard 1**: Patients referred to the NLC know the risk factors for chronic diseases, complications, and prevention methods.	37 (92.5)	2 (5.0)	0	39 (97.5)	40 (100)	0	0	40 (100)	38 (95.0)	2 (5.0)	0	40 (100)
	**Knowledge**		**Standard 2**: Patients referred to the NLC know how to improve and maintain a healthy lifestyle.	38 (95/0)	1 (2.5)	0	39 (97.5)	40 (100)	0	0	40 (100)	37 (92.5)	1 (2.5)	0	38 (95.0)
	**Application of knowledge**		**Standard 3**: The NLC clients make informed decisions to control their illness and lead a healthy lifestyle based on cultural and religious values ​​and socioeconomic status.	36 (90.0)	2 (5.0)	1 (2.5)	39 (97.5)	35 (87.5)	3 (7.5)	2 (5.0)	40 (100)	36 (90.0)	2 (5.0)	0	38 (95.0)
	**Clinical Outcomes**		**Standard 4**: The physical, mental, and emotional health of patients referred to the NLC is promoted.	37 (92.5)	1 (2.5)	1 (2.5)	39 (97.5)	39 (97.5)	0	0	39 (97.5)	38 (95.0)	2 (5.0)	0	40 (100)
	**Improving the performance of the NLCs**		**Standard 5**: The hospital examines the short-term and long-term consequences of education and counseling services to patients and their families.	37 (92.5)	1 (2.5)	1 (2.5)	39 (97.5)	39 (97.5)	1 (2.5)	0	40 (100)	37 (92.5)	2 (5.0)	0	39 (97.5)

## Discussion and conclusion

### Discussion

Using a mixed-method design, we developed practice standards for patient education in NLCs in Iran. The findings are likely to be helpful for both new and existing NLCs, as they can use them to evaluate their ongoing activities in light of the standard. This evaluation will contribute to the improvement of patient education in nurse-led clinics. Based on the review of documents and articles, we found no structure, process, or outcome standards for patient education in NLCs. Concerning other settings, most standards in the literature were related to the patient education process, and there was a need to develop standards for the structure and outcome domains. Also, the current process standards regarding referrals to other centers and patient follow-up are inadequate.

Concerning developing standards in this study, we defined the structure of NLCs for patient education in 6 domains (Table 4, Additional file 1) and the patient education process in four domains: (1) Organizational processes, (2) group processes, (3) individual training processes, and (4) the process of preparing educational content. Also, we defined the outcomes of patient education in 5 domains (Table 4, Additional file 1). In our context, one of the barriers to patient education is unsupportive organizational culture [38]. Developing NLCs need managerial support, development role, providing facility to play this role, control, and teamwork. Therefore, most agreements have been about standards related to the role of management.

Based on the results, there was the greatest level of agreement among the standards in the structure domain with standards 1 and 2, which discussed forming a working group/committee for patient education and the involvement of managers in setting up NLCs. During a change process, managers and employees are divided into two groups: Change agents (usually managers) and change recipients (usually employees). A change agent aims to identify strategies to facilitate the change process, while a change recipient aims to determine how the change directly impacts them [39]. Buick et al. (2018) confirmed that middle managers and leaders know their central roles in managing organizational changes. They interpret the communication from senior management regarding the changing intentions and translate it to clarify roles for employees, address the areas of resistance, and implement the changes [40].

Based on the results, the standards related to educational programs, materials, and content, methods of educating patients, and evaluating patients’ perception of education received the highest level of agreement in the process domain. Unlike verbal instructions, patient education materials serve as popular and permanent records of patient instructions [41]. Therefore, they should be accurate and include only treatments that are accepted in common practice. Patient education materials designed correctly and appropriately can augment other educational efforts and improve patient care [42]. Various methods can provide education, but direct interactions between the patient and the provider are perhaps the most common or face-to-face education. There is, however, evidence that written educational materials can help patients become more knowledgeable about medical conditions and possible treatments [43]. There is some evidence that written materials and other forms of patient education can significantly improve knowledge retention over time [44].

Regarding the outcome domain, the standards related to primary prevention and improving the performance of the NLC had the highest level of agreement. There are more deaths from chronic diseases than all other causes combined in developed and developing countries, accounting for approximately 43% of the global disease burden [45]. Approximately 60% of people over 65 have two or more chronic diseases [46]. There is a need for reforms to healthcare systems so that patients with multi-morbidity can access integrated, efficient, and effective healthcare [47]. Nurse-led clinics are especially ideal for preventing chronic diseases because patients and their families refer to such clinics, and primary prevention applies to the families. Improving the performance of the NLC can help with education and disease prevention in society.

### Conclusion

Following a well-established and clear methodology, we developed practice standards for patient education in NLCs. The standard inventory consisted of 46 statements in three domains (structure, process, and outcome), which might serve as a useful guide for clinical activities and a tool to assess the quality of patient education in NLCs. One of the strengths of this study was the participation of different groups of managers, service provider (physicians and nurses) and service recipients (patients and their care givers) in the development of the standard.

### Practice implications

Standard development for nursing practice can expand nursing roles and professionalism. Developed standards in this study can guide new and existing nurse-led clinics and help them evaluate ongoing activities, all of which contribute to improving patient education and performing safe and effective care in these clinics.

### Limitations

Our study had several limitations. First, most of our study participants were female because most nurses are female in our healthcare system. Second, at the beginning of the COVID-19 pandemic, the activities of NLCs were limited, and access to patients for interviews was difficult. However, all patients and caregivers participating in the qualitative phase were female; data saturation was the criterion for the end of sampling.

### Supplementary Information

**Additional file 1. **Developed Patient Education Standards for NLCs.

## Data Availability

All data generated or analyzed during this study are included in this published article [and its supplementary information files].

## References

[1] Randall S, Crawford T, Currie J, River J, Betihavas V (2017). Impact of community based nurse-led clinics on patient outcomes, patient satisfaction, patient access and cost effectiveness: a systematic review. Int J Nurs Stud.

[2] Hoare KJ, Mills J, Francis K (2012). The role of government policy in supporting nurse-led care in general practice in the United Kingdom, New Zealand and Australia: an adapted realist review. J Adv Nurs.

[3] Bentley M, Stirling C, Robinson A, Minstrell M (2016). The nurse practitioner–client therapeutic encounter: an integrative review of interaction in aged and primary care settings. J Adv Nurs.

[4] Rush KL, Burton L, Schaab K, Lukey A (2019). The impact of nurse-led atrial fibrillation clinics on patient and healthcare outcomes: a systematic mixed studies review. Eur J Cardiovasc Nurs.

[5] Connolly C, Cotter P. Effectiveness of nurse-led clinics on healthcare delivery: an umbrella review. J Clin Nurs. 2023;32(9-10):1760–7. 10.1111/jocn.16186. 10.1111/jocn.16186 34970816

[6] Olivia C, Hastie C, Farshid A (2021). Adherence to guidelines regarding anticoagulation and risk factors for progression of atrial fibrillation in a nurse-led clinic. Intern Med J.

[7] Hansen-Turton T, Sherman S, King ES (2016). Nurse-led health clinics: Operations, policy, and opportunities. J Nurs Regul.

[8] Schadewaldt V, Schultz T (2011). Nurse-led clinics as an effective service for cardiac patients: results from a systematic review. Int J Evidence‐Based Healthc.

[9] Hjorth M, Sjöberg D, Svanberg A, Kaminsky E, Langenskiöld S, Rorsman F (2018). Nurse-led clinic for patients with liver cirrhosis-effects on health-related quality of life: study protocol of a pragmatic multicentre randomised controlled trial. BMJ open.

[10] Piersma FR, Neefs J, Berger WR, van den Berg NWE, Wesselink R, Krul SPJ, de Groot JR. Care and referral patterns in a large, dedicated nurse-led atrial fibrillation outpatient clinic. Neth Heart J. 2022;30(7-8):370–6. 10.1007/s12471-021-01651-x. 10.1007/s12471-021-01651-x PMC9270511 34919210

[11] Ngcozana T, Ong VH, Denton CP (2020). Improving access to digital ulcer care through nurse-led clinic: a service evaluation. Musculoskelet Care.

[12] Wang Q, Shen Y, Chen Y, Li X (2019). Impacts of nurse-led clinic and nurse-led prescription on hemoglobin A1c control in type 2 diabetes: a meta-analysis. Medicine.

[13] Fishburn A, Fishburn N (2021). Establishing a nurse-led thyroid cancer follow-up clinic. Br J Nurs.

[14] Larsson I, Bergman S, Fridlund B, Arvidsson B (2012). Patients’ experiences of a nurse-led rheumatology clinic in Sweden: a qualitative study. Nurs Health Sci.

[15] Cheng HY, Chair SY, Wang Q, Sit JW, Wong EM, Tang SW (2016). Effects of a nurse-led heart failure clinic on hospital readmission and mortality in Hong Kong. J geriatric cardiology: JGC.

[16] Abraham CM, Norful AA, Stone PW, Poghosyan L (2019). Cost-effectiveness of Advanced Practice Nurses compared to Physician-Led Care for Chronic Diseases: a systematic review. Nurs Econ.

[17] Jones T, Shaban RZ, Creedy DK (2015). Practice standards for emergency nursing: an international review. Australasian Emerg Nurs J.

[18] Bastable SB. Nurse as educator: Principles of teaching and learning for nursing practice. 5th ed. United States of America: Jones & Bartlett Learning; 2019.

[19] Heshmai-Nabavi F, Mikaniki T, Ezati MH, Pouresmail Z (2015). Management of self-care education, from creation to regular monitoring: Mashhad University of Medical Sciences self-care education committee experience.

[20] Shirazi M, Anousheh M (2010). Review of history and changes in self care education to diabetic patients in the world, Iran and the nurses position in this field. Iran J Educ Med Sci.

[21] Douglas C, Schmalkuche D, Nizette D, Yates P, Bonner A (2018). Nurse-led services in Queensland: a scoping study. Collegian.

[22] Farahani M, Mohammadi E, Ahmadi F, Maleki M (2007). Cultural beliefs and behaviors of patients with coronary artery disorders: a necessity in patient education. Adv Nurs Midwifery.

[23] Announcement of executive. Instructions “Nursing clinics of education and follow-up” 2022[19-08-2023]. Available from: https://darman.sums.ac.ir/Dorsapax/userfiles/Sub132/Form/parastari/dastorolamal-ejraei-parastar-peygir-1402.pdf.

[24] Donabedian A (1969). Quality of care: problems of measurement. II: some issues in evaluating the quality of nursing care. Am J Public Health Nations Health.

[25] Donabedian A. The quality of care. How can it be assessed?. JAMA. 1988;260:1743e1748. 10.1001/jama.260.12.1743 3045356

[26] Sidani S, Doran DM, Mitchell PH (2004). A theory-driven approach to evaluating quality of nursing care. J Nurs Scholarsh.

[27] Thomas B, Gorospe G, Cooke L, Giesie P, J. Transitions of care: a hematopoietic stem cell transplantation nursing education project across the trajectory. Clin J of Oncol Nurs. 2015;19(4):E74–9. 10.1188/15.CJON.E74-E79 26207720

[28] Marquis BL, Huston CJ. Leadership roles and management functions in nursing: theory and application. 9th ed. Lippincott Williams & Wilkins; 2017. Printed in China.

[29] Creswell JW, Clark VLP (2017). Designing and Conducting Mixed Methods Research.

[30] Assarroudi A, Heshmati Nabavi F, Armat MR, Ebadi A, Vaismoradi M (2018). Directed qualitative content analysis: the description and elaboration of its underpinning methods and data analysis process. J Res nursing: JRN.

[31] Barber-Parker ED (2002). Integrating patient teaching into bedside patient care: a participant-observation study of hospital nurses. Patient Educ Couns.

[32] Assarroudi A, Heshmati Nabavi F, Armat MR, Ebadi A, Vaismoradi M (2018). Directed qualitative content analysis: the description and elaboration of its underpinning methods and data analysis process. J Res Nurs.

[33] Elo S, Kyngäs H (2008). The qualitative content analysis process. J Adv Nurs.

[34] Keeney S, McKenna HA, Hasson F. The Delphi technique in nursing and health research. United Kingdom: Wiley; 2011.

[35] Keeney S, Hasson F, McKenna H (2006). Consulting the oracle: ten lessons from using the Delphi technique in nursing research. J Adv Nurs.

[36] Drisko J, Maschi T (2015). Content. Analysis.

[37] 5-Point Likert Scale. In. In: Preedy VR, Watson RR, editors. Handbook of Disease Burdens and Quality of Life Measures. New York, NY: Springer New York; 2010. p. 4288.

[38] Karimi Moonaghi H, Emami Zeydi A, Mirhaghi A (2016). Patient education among nurses: bringing evidence into clinical applicability in Iran. Investigacion y educacion en enfermeria.

[39] Barratt‐Pugh L, Bahn S, Gakere E. Managers as change agents: Implications for human resource managers engaging with culture change. J Organizational Change Manage. 2013;26(4):748–64. 10.1108/JOCM-Feb-2011-0014.

[40] Buick F, Blackman D, Johnson S (2018). Enabling middle managers as change agents: why organisational support needs to change. Australian J Public Adm.

[41] Nikraftar F, Heshmati Nabavi F, Dastani M, Mazlom SR, Mirhosseini S (2022). Acceptability, feasibility, and effectiveness of smartphone-based delivery of written educational materials in iranian patients with coronary artery disease: a randomized control trial study. Health Sci Rep.

[42] Aldridge MD (2004). Writing and designing readable patient education materials. Nephrol Nurs J.

[43] Friedman AJ, Cosby R, Boyko S, Hatton-Bauer J, Turnbull G (2011). Effective teaching strategies and methods of delivery for patient education: a systematic review and practice guideline recommendations. J Cancer Educ.

[44] Wilson EA, Park DC, Curtis LM, Cameron KA, Clayman ML, Makoul G (2010). Media and memory: the efficacy of video and print materials for promoting patient education about asthma. Patient Educ Couns.

[45] World Health Organization. Integrated chronic disease prevention and control. 2017. Retrieved from http://www.who.int/chp/about/integratedcd/en/.

[46] Australian Institute of Health and Welfare (2016). Australia’s health 2016. Cat. No. AUS199.

[47] Bonner A, Havas K, Stone C, Abel J, Barnes M, Tam V (2020). A multimorbidity nurse practitioner-led clinic: evaluation of health outcomes. Collegian.

