# Supplementary material for: The development of practice standards for patient education in nurse-led clinics: a mixed-method study
Source: BMC Nurs. 2023 Aug 22;22:277. doi: 10.1186/s12912-023-01444-0 (PMC10464384; doi:10.1186/s12912-023-01444-0)
Supplement: Supplementary file 1 — Additional file 1. Developed Patient Education Standards for NLCs. [file 12912_2023_1444_MOESM1_ESM.docx]

**Additional file 1: Developed Patient Education Standards for NLCs**

| **Domain** | | | **standards** |
| --- | --- | --- | --- |
| **Structure** | **team/ teamwork** | | **Standard 1:** The head and director of the hospital, the director of nursing, the health education supervisor, and the head nurse of the clinic cooperate in establishing and supervising the Nurse-led Clinic (NLC). |
|  |  |  | **Standard 2:** The patient education committee in the hospital has been formed with the participation of the head and director of the hospital, the nursing director, the health education supervisor, the head nurse of the clinic, and educating nurses in the NLC. |
|  |  |  | **Standard 3:** The hospital has defined the mechanism of interdisciplinary cooperation in patient and family education in the NLC. |
|  |  |  | **Standard 4:** The hospital has specified and announced the role and duties of the nurse, physician, and non-professional staff of the clinic (secretary, guard, etc.) regarding the activities of the NLC. |
|  |  |  | **Standard 5:** A job description exists and is available for the educating nurse in the NLC. |
|  | **Advisory Committee** | | **Standard 6:** The hospital provides counseling services for nursing educators in patient education (the possibility of contracting and consulting with medical and nursing professors, books, and updated print and online instructions) to answer patients' questions. |
|  | **Professional skills and continuing education** | | **Standard 7:** The hospital has provided the possibility of participating nursing educators in the NLC in codified patient education courses, health literacy, self-care, and self-management. |
|  |  |  | **Standard 8:** The hospital selects educating nurses in the NLC based on their competencies. |
|  |  |  | **Standard 9:** The hospital selects the educating nurses in the NLC based on their meta-competencies. |
|  | **Physical space and equipment, Facilities, and planning** | | **Standard 10:** The hospital provides the standard physical environment for the NLC. |
|  |  |  | **Standard 11:** The hospital provides training equipment, facilities, and educational assistance tools based on patients' and their families' educational needs and preferences. |
|  |  |  | **Standard 12:** The hospital has provided the necessary facilities for patients to access the NLC. |
|  |  |  | **Standard 13:** In the hospital's operational plan, planning has been done to develop training and counseling services in the NLC. |
| **Process** | **1. Organizational processes** | **patient education** | **Standard 1:** The hospital determines the components of the patient education process, including needs assessment, planning, implementation, and evaluation of education. |
|  |  | **Audience training and referral** | **Standard 2:** Recipients of services in the NLC are determined based on the type of disease and the number of patients referred to hospital outpatient clinics. |
|  |  |  | **Standard 3:** The hospital uses appropriate and effective methods to introduce the services of the NLC, identify patients needing training and counseling, and refer them to the NLC. |
|  |  |  | **Standard 4:** If necessary, educating nurses in the NLC and considering the patient and family preferences refers them to the NLC in specialized and sub-specialized hospitals and related social organizations. |
|  |  | **Clinic activity time** | **Standard 5:** The hospital provides patients with access to the education and counseling services of the NLC at the appropriate time with a minimum increase in waiting time and in an appropriate manner. |
|  |  | **Decision making, and problem-solving** | **Standard 6:** The hospital plans to improve the quantity and quality of services in the NLC. |
|  |  |  | **Standard 7:** Education and counseling services are planned and implemented based on the type of the hospital in which lifestyle-related chronic diseases (coronary artery disease, hypertension, diabetes, and cancer) have a priority for the elderly, pregnant women, and children. |
|  |  |  | **Standard 8:** Based on a pre-designed operational plan, the activities of the NLC are performed and monitored. |
|  |  |  | **Standard 9:** The hospital provides appropriate facilities and incentives to encourage patients and their families to visit the NLC. |
|  |  |  | **Standard 10:** The hospital supports creative and innovative methods to remove barriers to patient education in the NLC. |
|  | **2. Group processes** | **Needs assessment** | **Standard 11:** The needs and educational priorities of patients referred to the NLC are properly determined at appropriate intervals in the hospital. |
|  |  | **Determining learning objectives and designing a patient education program** | **Standard 12:** A codified educational program for common diagnoses referred to as the NLC is planned, implemented, and evaluated with a precise definition of learning objectives and training schedules. |
|  |  | **Educational content and use of educational materials** | **Standard 13:** Patients and families are educated concerning specific care measures, nutrition and diet therapy, drug use, self-care training, joint care, physical activity, disease screening, mental health, personal hygiene, maternal health, smoking cessation, rehabilitation, safe use of the equipment, and home safety. |
|  |  |  | **Standard 14:** Educating nurses use appropriate educational materials (pamphlets, posters, videos, pictures, diagrams, and forms during discharge) to educate and strengthen patient and family learning based on their needs and preferences. |
|  |  | **Recording patient education** | **Standard 15:** The hospital uses the appropriate methods to record patient education reports (including learning needs, learners, training method, duration of the training, and feedback upon training). |
|  |  |  | **Standard 16:** Recording patient education should be accurate, precise, and legal. |
|  |  | **evaluation** | **Standard 17:** The hospital evaluates NLC programs and reports their results to stakeholders. |
|  |  |  | **Standard 18:** Codified training programs are reviewed at appropriate intervals. |
|  |  |  | **Standard 19:** The hospital plans and takes corrective measures to evaluate the quality and appropriateness of educational materials (educational texts and videos). |
|  |  | **patient Follow-up** | **Standard 20:** The hospital plans to provide post-discharge education and follow-up services for at least three groups of priority patients. |
|  | **3. Individual training processes** | **Needs assessment and training prioritization** | **Standard 21:** The educating nurse conducts a needs assessment, determines the patient and family's readiness and learning styles, and records the results. |
|  |  |  | **Standard 22:** Educational needs are prioritized based on individual needs assessment and a well-designed program. |
|  |  | **method and duration of the training** | **Standard 23:** Teaching patients in the NLC involves the combination of face-to-face and online methods, considering patients' and families' facilities, values ​​, and preferences. |
|  |  |  | **Standard 24:** Patient education in the NLC includes privacy, confidentiality, and respect for patient's values ​​and opinions. |
|  |  |  | **Standard 25:** Patient education in the NLC is planned and implemented as soon as possible, considering the patient's physical condition and avoiding wasting time. |
|  |  | **Evaluate patient education** | **Standard 26:** At the end of the training session, the teaching nurse assesses the understanding of the patient and the family from the education by questioning the patients and observation and representation methods and records the results in the patient education registration form in the NLC or the patient's electronic file. |
|  |  | **The process of preparing educational content** | **Standard 27:** The hospital plans to prepare and update appropriate educational materials with quality based on the needs and preferences of patients. |
|  |  |  | **Standard 28:** The hospital uses appropriate methods for distributing and storing educational content and materials (pamphlets and multimedia). |
| **Outcome** | **Primary prevention** | | **Standard 1:** Patients referred to the NLC know the risk factors for chronic diseases, complications, and prevention methods. |
|  | **Knowledge** | | **Standard 2:** Patients referred to the NLC know how to improve and maintain a healthy lifestyle. |
|  | **Application of knowledge** | | **Standard 3:** The NLC clients make informed decisions to control their illness and lead a healthy lifestyle based on cultural and religious values ​​and socioeconomic status. |
|  | **Clinical Outcomes** | | **Standard 4:** The physical, mental, and emotional health of patients referred to the NLC is promoted. |
|  | **Improving the performance of the NLCs** | | **Standard 5:** The hospital examines the short-term and long-term consequences of education and counseling services to patients and their families. |
